# Temporal relationship of sleep apnea and acromegaly: a nationwide study

**DOI:** 10.1007/s12020-018-1694-1

**Published:** 2018-07-31

**Authors:** Konstantina Vouzouneraki, Karl A. Franklin, Maria Forsgren, Maria Wärn, Jenny Tiberg Persson, Helena Wik, Christina Dahlgren, Ann-Sofie Nilsson, Caroline Alkebro, Pia Burman, Eva-Marie Erfurth, Jeanette Wahlberg, Anna-Karin Åkerman, Charlotte Høybye, Oskar Ragnarsson, Britt Edén Engström, Per Dahlqvist

**Affiliations:** 1 Department of Public Health and Clinical Medicine, Umeå University, Umeå, Sweden; 2 Department of Surgical and Perioperative Sciences, Umeå University, Umeå, Sweden; 3 Department of Medical Sciences, Endocrinology and Mineral Metabolism, Uppsala University, Uppsala, Sweden; 4 Department of Molecular Medicine and Surgery, Patient Area Endocrinology and Nephrology, Inflammation and Infection Theme, Karolinska Institute and Karolinska University Hospital, Stockholm, Sweden; 5 Department of Endocrinology, Institute of Medicine, Sahlgrenska Academy, University of Gothenburg and Sahlgrenska University Hospital, Gothenburg, Sweden; 6 Department of Endocrinology, Department of Medical and Health Sciences, Department of Clinical and Experimental Medicine, Linköping University, Linköping, Sweden; 7 Department of Clinical Sciences and Department of Endocrinology, University of Lund and Skåne University Hospital, Malmö - Lund, Sweden; 8 Division of Diabetology and Endocrinology, Department of Medicine, Örebro University Hospital, Örebro, Sweden

**Keywords:** Acromegaly, Sleep apnea, Comorbidities, Risk factors

## Abstract

**Purpose:**

Patients with acromegaly have an increased risk of sleep apnea, but reported prevalence rates vary largely. Here we aimed to evaluate the sleep apnea prevalence in a large national cohort of patients with acromegaly, to examine possible risk factors, and to assess the proportion of patients diagnosed with sleep apnea prior to acromegaly diagnosis.

**Methods:**

Cross-sectional multicenter study of 259 Swedish patients with acromegaly. At patients’ follow-up visits at the endocrine outpatient clinics of all seven university hospitals in Sweden, questionnaires were completed to assess previous sleep apnea diagnosis and treatment, cardiovascular diseases, smoking habits, anthropometric data, and S-IGF-1 levels. Daytime sleepiness was evaluated using the Epworth Sleepiness Scale. Patients suspected to have undiagnosed sleep apnea were referred for sleep apnea investigations.

**Results:**

Of the 259 participants, 75 (29%) were diagnosed with sleep apnea before the study start. In 43 (57%) of these patients, sleep apnea had been diagnosed before the diagnosis of acromegaly. After clinical assessment and sleep studies, sleep apnea was diagnosed in an additional 20 patients, yielding a total sleep apnea prevalence of 37%. Higher sleep apnea risk was associated with higher BMI, waist circumference, and index finger circumference. Sleep apnea was more frequent among patients with S-IGF-1 levels in the highest quartile.

**Conclusion:**

Sleep apnea is common among patients with acromegaly, and is often diagnosed prior to their acromegaly diagnosis. These results support early screening for sleep apnea in patients with acromegaly and awareness for acromegaly in patients with sleep apnea.

## Introduction

Acromegaly is a rare disease with an annual incidence of 3–7 cases per 1 million persons, and a prevalence of 60–134 per million [1–5]. It is almost always caused by a growth hormone-secreting pituitary adenoma [4, 6]. Patients with acromegaly show increased risks of hypertension, cardiomyopathy, and diabetes mellitus and have a 2-fold excess mortality rate [1, 6–10]. Acromegaly is often diagnosed several years after the initial onset of symptoms, by which point most patients have marked and irreversible disease manifestations [11, 12]. Such diagnostic delays increase mortality and allow further pituitary adenoma growth that reduces the possibilities for complete surgical excision [4, 6, 11].

Patients with acromegaly also often suffer from sleep apnea [9, 10, 13–22], which is characterized by recurrent episodes of apnea during sleep followed by hypoxemia, sympathetic activation, frequent arousals from sleep, and excessive daytime sleepiness [23, 24]. The most common form is obstructive sleep apnea, in which the upper airways are obstructed during sleep due to enlargement of craniofacial, pharyngeal, and laryngeal tissue [7, 16]. Sleep apnea is associated with hypertension, stroke, and premature death [14, 16, 25–27]. Thus, it may be hypothesized that sleep apnea contributes to decreased quality of life, comorbidity, and mortality in acromegaly, warranting increased attention to this complication [7]. Previous studies report highly variable prevalence of sleep apnea among patients with acromegaly (11–87%), with higher prevalence in smaller prospective studies using polysomnography in all patients and lower in large retrospective epidemiological studies [9, 10, 13, 15–22, 28–30]. According to a recent Italian study of the awareness and management of sleep apnea in acromegaly, 43% of centers referred less than 20% of their patients for polysomnography, raising concerns about possible under-diagnosis of the disorder [31].

Here we aimed to estimate the prevalence of sleep apnea in a large national cohort of patients with acromegaly, as well as to evaluate risk factors in this population. We also assessed the temporal relationship between diagnosis of sleep apnea and acromegaly in patients with both conditions, and evaluated whether the use of a simple screening instrument at the endocrine outpatient clinic could improve sleep apnea diagnosis in patients with acromegaly.

## Subjects and methods

### Patients

This study included patients who were diagnosed with acromegaly in 1991 or later. Patients were invited to participate during routine follow-up visits at endocrine outpatient clinics of all seven Swedish university hospitals between January 2013 and January 2015. This study also included eight patients from a pilot study conducted using the same protocol at Umeå University Hospital between November 2008 and June 2011. Inclusion criteria were confirmed acromegaly diagnosis and consent to participate. There were no exclusion criteria.

The study visits occurred at a median of 7.7 years (range, 0–23 years) after acromegaly diagnosis. Following a standardized protocol, patients and physicians completed questionnaires (Online Resource 2 and Online Resource 3) with information about snoring, witnessed apneas during sleep, smoking habits, previous sleep apnea investigations and treatments, and acromegaly treatment. Questions regarding previous myocardial infarction and stroke, as well as current treatment for hypertension, diabetes mellitus, heart failure, and angina pectoris were also included. Daytime sleepiness was assessed using the Epworth Sleepiness Scale (ESS). Measurements were taken of height, weight, blood pressure, index finger circumference, and waist circumference.

We also acquired information regarding the date of diagnosis and previous treatments for acromegaly from the Swedish Pituitary Registry and from medical records when necessary. The Swedish Pituitary Registry has compiled information on clinical findings, biochemical analyses, surgical, medical, and radiation treatments, and outcomes in patients with pituitary adenomas, including acromegaly since 1991.

### Sleep apnea investigations

Patients with daytime sleepiness (ESS ≥ 10) or clinical suspicion of sleep apnea based on snoring and partner-observed apnea were referred for overnight sleep apnea investigations using the Embletta or Embla systems. They included continuous recordings of oro-nasal airflow, respiratory effort, and oxygen saturation. Embla polysomnographic investigations also included electroencephalograms (EEG), chin electromyograms (EMG), and electrooculograms (EOG). The results of all sleep apnea investigations were collected and assessed by a specialist in sleep medicine and respiratory medicine (KF), who contacted local sleep apnea clinics and reassessed the sleep studies when necessary. Sleep apnea was classified based on the apnea-hypopnea index, i.e., the mean number of apneas and hypopneas per hour of sleep. An apnea-hypopnea index of 5–15 was classified as mild sleep apnea, 15–30 indicated moderate sleep apnea, and >30 indicated severe sleep apnea.

### S-IGF-1 and biochemical control of acromegaly

Venous blood was drawn before each patient’s visit. Serum levels of insulin-like growth factor 1 (S-IGF-1) were measured at the accredited laboratory at each university hospital. S-IGF-1 levels were assessed relative to the reference interval for the patient’s age, and are thus presented as the percentage of the upper limit of normal (ULN) for the patient’s age. We defined biochemical control as an S-IGF-1 level of less than or equal to the ULN for the patient’s age.

### Statistics

Descriptive data are presented as proportions or percentages. We used the chi-square test to assess potential associations between sleep apnea and dichotomous risk factors (i.e., gender and smoking), and to compare sleep apnea frequency between patients with S-IGF-1 values in the highest quartile (Q4) versus in the other quartiles (Q1–Q3). To calculate the odds ratio (OR) for sleep apnea relative to risk factors with continuous variables (i.e., age, S-IGF-1, BMI, etc.), we performed univariate logistic regression, followed by multivariable logistic regression analysis (i.e., for each risk factor including age and gender as covariates). The results of the age-adjusted and gender-adjusted regressions are presented as OR for sleep apnea with a 95% confidence interval (CI). All calculations were performed using SPSS v 23 for Macintosh (IBM). A *p* value of <0.05 was considered significant.

## Results

### Patients characteristics reported at study start

**Fig. 1:**
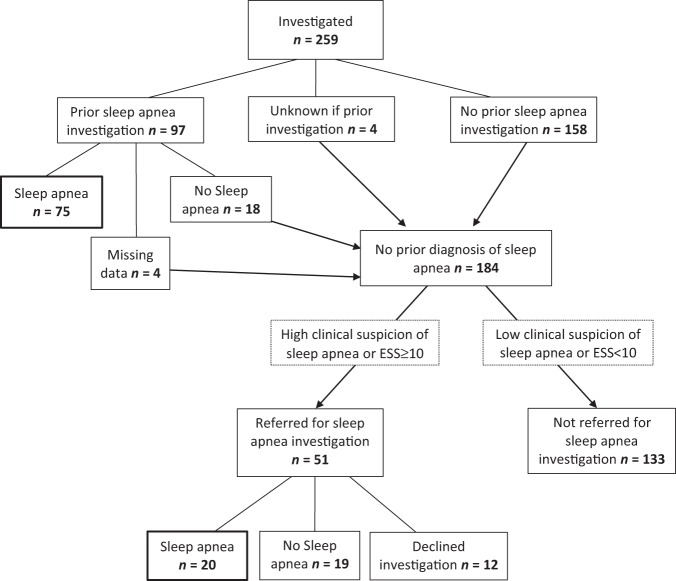
Flow diagram showing the number of patients in each group that emerged during the study process

**Fig. 2:**
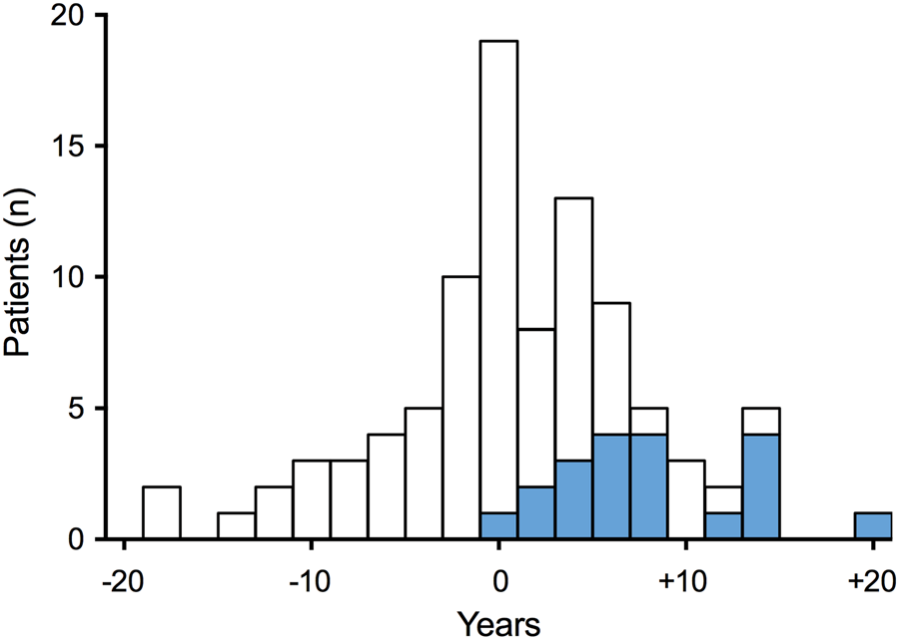
Diagnosis of sleep apnea in relation to the diagnosis of acromegaly (0 years). White bars □ represent the 75 patients with sleep apnea diagnosed prior to studyBlue bars  represent the 20 patients with sleep apnea identified in the study

This study investigated 259 patients with acromegaly, 127 women and 132 men. Background characteristics are presented in Table 1. Of the 259 patients, 97 (37%) had previously undergone a sleep apnea investigation, and 75/97 (77%) had been diagnosed with sleep apnea (Fig. 1). Of these 75 patients 35 (47%) were treated with continuous positive airway pressure (CPAP) and 11 (15%) with mandibular advancement splint. Among the patients with previously diagnosed sleep apnea, 43/75 (57%) were diagnosed with sleep apnea prior to their diagnosis with acromegaly (Fig. 2). Thus, of the total 259 participants, 43 (17%) were diagnosed with sleep apnea before being diagnosed with acromegaly. Within this group, the mean duration from sleep apnea diagnosis to acromegaly diagnosis was 4 years (median, 2 years; range, 0–18 years) (Fig. 2).

**Table 1:** Background characteristics of the 259 patients with acromegaly

Female/male (*n*)	127/132
Age in years, mean ± SD (range)	57 ± 13 (19–87)
Age at acromegaly diagnosis in years, mean ± SD (range)	48 ± 13 (19–80)
Current medical treatment for acromegaly, *n* (%)	80 (31)
Pituitary surgery, *n* (%)	231 (89)
Pituitary radiotherapy, *n* (%)	46 (18)
Biochemical control (S-IGF-1 ≤ ULN), n (valid %, *n* = 258)	177 (69)
Current smokers, *n* (%)	41 (16)
BMI, mean ± SD	29 ± 5

### Clinical assessment and sleep apnea investigation during the study

Of the 184 patients without a prior sleep apnea diagnosis, 51 (28%) were offered a sleep apnea investigation due to high clinical suspicion of sleep apnea at the study visit. Twenty-two of these patients had ESS < 10 but still considered to have a high clinical suspicion of sleep apnea and were therefore referred to a sleep apnea investigation. On the other hand, 7 patients with ESS ≥ 10 were not referred to a sleep investigation due to low clinical suspicion of sleep apnea. The patients with high clinical suspicion of sleep apnea (*n* = 51) had a median ESS of 10, compared to the median ESS of 4 among the patients with low clinical suspicion of sleep apnea (*n* = 133) (*p* *<* 0.001, Mann–Whitney). Of these 51 patients, 12 declined and 39 underwent sleep apnea investigations. Sleep apnea was diagnosed in 20 patients (51%), with 10 having mild sleep apnea, 5 moderate sleep apnea, and 5 severe sleep apnea. Thus, of the 184 patients without previously known sleep apnea, 20 (11%) were diagnosed with sleep apnea in the current study. Overall, of the 259 patients with acromegaly, 136 (53%) were investigated for sleep apnea either before or during the study, and 95 (37%) were diagnosed with sleep apnea either before (*n* = 75) or during the study (*n* = 20) (Fig. 1). Table 2 summarizes the characteristics of the patients with sleep apnea (*n* = 95) and the patients not diagnosed with sleep apnea (*n* = 164) in our cohort.

**Table 2:** Characteristics of acromegaly- patients with, and without previously or newly diagnosed sleep apnea

	Sleep apnea (*n* = 95)	No sleep apnea (*n* = 164)	*p* value
Age in years^a^	59 (53–67)	58 (45–68)	0.14
Years since acromegaly diagnosis^a^	6.5 (2.9–12.5)	8.4 (4.0–14.7)	0.072
Men^b^ (%)	55	49	0.36
Current smokers^b^, valid %, *n* = 255	15	17	0.65
Current or previous smokers^2^, valid %, *n* = 255	51	46	0.45
Snoring^b^, valid %, *n* = 253	62	43	0.009
Partner-observed apneas during sleep^b^, valid %, *n* = 257	44	15	<0.001
BMI^a^ (kg/m^2^)	30 (27–34)	26 (24–30)	<0.001
Waist circumference^a^ (cm)	104 (92–113)	95 (86–104)	<0.001
Index finger circumference^a^ (mm)	75 (69–81)	71 (68–79)	0.006
S-IGF-1 in the highest quartile^b^	33	20	0.021
Hypertension, current treatment^b^ (%)	53	46	0.28
Diabetes, current treatment^b^ (%)	8.4	8.5	0.97
Stroke or TIA^b^ (%)	3.2	6.7	0.22

^a^Data are presented as median (25th–75th percentile) and compared by Mann–Whitney test

^b^Data are presented as percentage and compared by chi square test

### Risk factors for sleep apnea in patients with acromegaly

Among patients with acromegaly, the risk for sleep apnea (previously or newly diagnosed) was significantly increased in patients with higher BMI, waist circumference, and index finger circumference (Table 3). In the logistic regression, S-IGF-1% (of ULN) was not significantly associated with the risk of sleep apnea (OR, 1.004; 95% CI, 0.999–1.009; *p* *=* 0.091). However, patients with S-IGF-1 in the highest quartile (Q4; corresponding to >1.09 × ULN) showed a significantly higher risk of sleep apnea (48%) compared to patients with S-IGF-1 in Q1–Q3 (32%) (*p* *=* 0.021). Sleep apnea risk was not significantly associated with age (*p* *=* 0.105), gender (*p* *=* 0.36), or smoking (*p* *=* 0.65) (Table 2).

**Table 3:** Odds ratio for sleep apnea with regards to BMI, waist circumference, and index finger circumference

Parameter	BMI (kg/m^2^)	Waist circumference (cm)	Index finger circumference (mm)
Odds ratio^a^	1.112	1.042	1.081
95% confidence interval	1.055–1.171	1.019–1.066	1.028–1.136
*p* value	<0.001	<0.001	0.002

^a^The odds ratio for sleep apnea was assessed using multivariable logistic regressions, with sleep apnea (previous or newly diagnosed) as the dependent variable and each risk factor as an independent variable (including age and sex as covariates)

### Hypertension and cardiovascular disease

Of the 259 patients with acromegaly, 125 (48%) exhibited hypertension. The prevalence of hypertension did not significantly differ between the groups of acromegaly patients with and without diagnosed sleep apnea (Table 2). The prevalence of cardiovascular diseases was low, with angina pectoris in 0.8%, congestive heart failure in 1.2%, previous myocardial infarction in 1.2%, and stroke or TIA in 5.4% of the patients (Online Resource 1). Therefore, no comparisons between patients with and without sleep apnea was made.

## Discussion

In this large national cohort of patients with acromegaly, 37% had sleep apnea. The diagnosis of sleep apnea occurred before acromegaly diagnosis in 17% of the patients, after acromegaly diagnosis in 12%, and after a simple systematic screening in another 8% of the patients. This is, to our knowledge, the first study to investigate the temporal relationship between sleep apnea diagnosis and acromegaly diagnosis. We further identified high BMI, waist circumference, and index finger circumference as significant risk factors for sleep apnea, and found that patients with S-IGF-1 in the highest quartile were more likely to have sleep apnea.

Previous prospective studies where all patients underwent polysomnography report sleep apnea prevalence rates ranging from 44–87% in patients with active acromegaly, and 35–58% among patients with biochemical control [9, 10, 13, 15–22]. In contrast, in large retrospective studies in which sleep apnea is assessed based on diagnosis codes (WHO ICD) in registry data, the reported sleep apnea prevalence ranges from 11–30% in patients with acromegaly (biochemical control not specified) [28–30]. The prevalence in our cohort was higher than in previous retrospective studies [28–30], but somewhat lower than in most previous prospective studies [9, 13, 15, 16, 18–22, 32, 33]. Our study included patients with treated acromegaly, with two-thirds in biochemical control, which could explain why the prevalence was most comparable to the rates in previous studies of patients with biochemically controlled acromegaly [13, 18, 19, 22]. A weakness of this study was that some patients with clinical suspicion of sleep apnea declined investigation and no investigations were made in patients with low clinical suspicion, i.e., low ESS and no snoring. This may have resulted in an underestimation of the prevalence of sleep apnea in our study.

Acromegaly is a rare disease and the phenotypic changes develop slowly. Together, these factors often contribute to unfortunate delays in diagnosis and treatment [34, 35]. Increased awareness of acromegaly in high-risk populations might reduce this diagnostic delay. Current guidelines recommend S-IGF-1 measurement in patients with several acromegaly-associated conditions, including sleep apnea, type 2 diabetes mellitus, debilitating arthritis, carpal tunnel syndrome, hyperhidrosis, and hypertension [6]. Previous studies report relatively higher prevalence of acromegaly among patients with diagnosed sleep apnea (135–350 per 100,000) than in the general population (4–13 per 100,000) [36–38]. In our study, acromegaly diagnosis was delayed by a mean of 4 years in 43 patients with known sleep apnea, supporting the possibility that acromegaly might be recognized earlier by high awareness or even screening for acromegaly at sleep clinics. However, due to the rarity of acromegaly further cost-effectiveness studies are needed to determine which patients with sleep apnea should be screened for acromegaly.

Effective acromegaly treatment reduces sleep apnea prevalence and severity [9, 15, 16, 19, 39]. Longitudinal studies report that biochemical control of acromegaly is associated with improvement of sleep apnea, possibly due to reduced soft tissue swelling and decreased chemosensitivity to hypoxia [9, 13, 15, 17, 19]. However, sleep apnea persists in at least 40% of the acromegaly patients after biochemical control and the need for treatment for sleep apnea often remains [13, 16, 17, 19, 39]. Contrary, there are also studies reporting no sleep apnea improvement after acromegaly treatment and no significant association (or even discordance) between hormonal status (S-IGF-1, S-GH) and sleep parameters after treatment [15, 32, 39]. In our current study, patients with S-IGF-1 in the highest quartile (Q4, corresponding to 1.09 × ULN) showed a significantly higher sleep apnea risk than patients in Q1–3. This suggested higher sleep apnea risk in patients whose acromegaly was clearly not biochemically controlled, in accordance with previous reports showing an association between sleep apnea and biochemical disease activity [10, 13, 17, 19, 22]. The finding that larger index finger circumference was a significant risk factor for sleep apnea further support the association between biochemical activity and sleep apnea. It is difficult to define biochemical control in acromegaly. According to the ACRODAT study, a S-IGF-1 level >1.2 × ULN per se should be considered to indicate “significant disease activity”, while other parameters should be considered before taking clinical action in cases with lower S-IGF-1 levels [40].

High BMI and obesity are well-known risk factors for sleep apnea among patients with acromegaly, as well as in the general population [10, 17, 19, 22, 23, 41, 42]. However, several smaller studies do not show an association between BMI and sleep apnea [9, 10, 13, 20, 33]. Among acromegaly patients, higher BMI might reflect both obesity and increased body water and fat-free mass due to poor biochemical control, both of which may impact sleep apnea. The present study confirmed that higher BMI and waist circumference both were associated with a significantly increased risk of sleep apnea (Table 2). Moreover, age, male gender, and smoking were not risk factors for sleep apnea in the present cohort, which is in accordance with some previous studies but in contrast to others [9, 10, 13, 17, 19, 22, 32, 33].

Hypertension is common in acromegaly and in sleep apnea [6, 8, 25]. In the current study 48% of acromegaly patients had treatment for hypertension. Hypertension was not more common in the acromegaly patients with diagnosed sleep apnea. However, it has to be noted that a large proportion of these patients had treatment for sleep apnea. No firm conclusions could be drawn regarding the other cardiovascular diseases attributable to sleep apnea due to the low prevalence of those diseases in our study population (Table 2, Online resource 1).

## Conclusions

In this large national study, we found that sleep apnea occurred in at least 37% of patients with acromegaly and many patients were diagnosed with sleep apnea before the diagnosis of acromegaly. Our results support that sleep apnea should be considered in all patients with acromegaly, especially among those with poor biochemical control or high BMI. We also suggest vigilance for acromegaly among patients with sleep apnea, since sleep apnea may be an early sign of acromegaly.

## Electronic supplementary material

Supplementary information

Supplementary information
