# Supplementary material for: Temporal relationship of sleep apnea and acromegaly: a nationwide study
Source: Endocrine. 2018 Jul 31;62(2):456–63. doi: 10.1007/s12020-018-1694-1 (PMC6208862; doi:10.1007/s12020-018-1694-1)
Supplement: Supplementary file 1 — Supplementary information [file 12020_2018_1694_MOESM1_ESM.pdf]

Temporal relationship of sleep apnea and acromegaly: a nationwide study

Endocrine

Konstantina Vouzouneraki*, Karl A. Franklin, Maria Forsgren, Maria Wärn, Jenny Tiberg Persson, Helena Wik, Christina Dahlgren, Ann-Sofie Nilsson, Caroline Alkebro, Pia Burman, Eva-Marie Erfurth, Jeanette Wahlberg, Anna-Karin Åkerman, Charlotte Høybye, Oskar Ragnarsson, Britt Edén Engström, Per Dahlqvist

* Corresponding author, Department of Public Health and Clinical Medicine, Umeå University
e-mail: konstantina.vouzouneraki@umu.se

Online Resource 1 Characteristics of 95 patients with newly or previously diagnosed sleep apnea and 164 patients without sleep apnea.

	**Sleep apnea (*n* = 95)**	**No sleep apnea (*n* = 164)**	**All (*n* = 259)**
Age in years*	59 (53–67)	58 (45–68)	59 (47–67)
Men, %	55	49	51
Current smokers, valid %, n = 255	15	17	16
Current or previous smokers, valid %, n = 255	51	46	47
Snoring, valid %, n = 253	62	43	50
Partner-observed apneas during sleep, valid %, n = 257	44	15	26
BMI*, kg/m^2	30 (27–34)	26 (24–30)	28 (25–32)
BMI <25 kg/m^2, valid %, n = 258	16	36	29
BMI 25–30 kg/m^2, valid %, n = 258	36	38	37
BMI >30 kg/m^2, valid %, n = 258	48	26	34
Waist circumference*, cm	104	95	98

	(92–113)	(86–104)	(87–109)
S-IGF-1, % of ULN *	86<br>(66–120)	78<br>(63–104)	82<br>(63–109)
S-IGF-1 ≤ULN, valid %, n = 258	64	71	69
S-IGF-1 in the highest quartile (Q4), %	33	20	25
Hypertension, current treatment, %	53	46	48
Diabetes, current treatment, %	8.4	8.5	8.5
Heart failure, current treatment, valid% n = 258	0	1.8	1.2
Angina Pectoris, current treatment, %	1.1	0.6	0.8
Myocardial infarction, previous, %	1.1	1.2	1.2
Stroke or TIA, previous, %	3.2	6.7	5.4
Index finger circumference*, mm	75<br>(69–81)	71<br>(68–79)	74<br>(68–80)
Years since acromegaly diagnosis*	6.5<br>(2.9–12.5)	8.4<br>(4.0–14.7)	7.7<br>(3.6–13.7)
Pituitary surgery, %	85	92	89
Pituitary radiotherapy, %	18	18	18
Current medical therapy for acromegaly, %	37	27	31
Somatostatin analogue, %	34	23	27
Dopamine agonist, %	2.1	3.7	3.1
Pegvisomant, %	8.4	7.9	8.1

* median (25th–75th percentile)
