# Supplementary material for: Temporal relationship of sleep apnea and acromegaly: a nationwide study
Source: Endocrine. 2018 Jul 31;62(2):456–63. doi: 10.1007/s12020-018-1694-1 (PMC6208862; doi:10.1007/s12020-018-1694-1)
Supplement: Supplementary file 2 — Supplementary information [file 12020_2018_1694_MOESM2_ESM.pdf]

Temporal relationship of sleep apnea and acromegaly: a nationwide study

Endocrine

Konstantina Vouzouneraki*, Karl A. Franklin, Maria Forsgren, Maria Wärn, Jenny Tiberg Persson, Helena Wik, Christina Dahlgren, Ann-Sofie Nilsson, Caroline Alkebro, Pia Burman, Eva-Marie Erfurth, Jeanette Wahlberg, Anna-Karin Åkerman, Charlotte Høybye, Oskar Ragnarsson, Britt Edén Engström, Per Dahlqvist

* Corresponding author, Department of Public Health and Clinical Medicine, Umeå University

e-mail: konstantina.vouzouneraki@umu.se

Online Resource 2 and Online Resource 3

Online Resource 2 The questionnaire completed by the patient at the study visit.

Questionnaire for the patients

Name :____

Personal identification number:____

Date of visit :______________________________________

	Yes	No	I don't know
1. Do you snore?	O	O	O
2. Have you noticed breathing cessations during sleep?	O	O	O
3. Do you feel rested after a nights sleep?	O	O	
4. Have you ever been investigated for sleep apnea?	O	O	O
a. If yes, when (year) and where? ____			
b. Did the investigation show sleep apnea?	O	O	O

c. If yes, do you have a treatment for sleep apnea? O CPAP O mandibular splint O Other:___________

Rate your probability of falling asleep in the following situations:

<u>**0** = would never doze, **1** = *slight chance of dozing*, **2** = *moderate chance of dozing*, **3** = *high chance of dozing*</u>

	0	1	2	3
Sitting and reading	O	O	O	O
Watching TV	O	O	O	O
Sitting inactive in a public place (meeting, theatre, etc.)	O	O	O	O
As a passenger in a car for 1 hour without a break	O	O	O	O
Lying down to rest in the afternoon	O	O	O	O
Sitting and talking to someone	O	O	O	O
Sitting quietly after a lunch without alcohol	O	O	O	O
In a car, while stopped for a few minutes	O	O	O	O

Epworth Sleepiness Scale (ESS)

Score ≥10 is associated with sleep apnea, lower score does not exclude it. Score: _______points

Online resource 3. The questionnaire completed by the patient's treating endocrinologist and nurse during the study.

Questionnaire for the treating doctor and nurse

Name: ____

Personal identification number: ____

Patient's identification for the study ________________________

Date of birth: 19__/__/__ O Male O Female

Has given written informed consent O No O Yes

Date of acromegaly diagnosis: __________________

Pituitary surgery O No OYes x1 OYes x2 OYes x>2

Pituitary radiotherapy O No O Yes

	**Yes/No**	**(year, month)**
• Has the patient had a myocardial infarction?	Y / N	_________
• Has the patient had a stroke?	Y / N	_________
• Is the patient smoking?	Y / never / quitted year	_________
• Current medical treatment for acromegaly?	Y / N	
O Somatostatin analogue O Dopamine agonist O Pegvisomant		
• Has the patient current medical treatment for hypertension?	Y / N	
• Has the patient current medical treatment for diabetes?	Y / N	
• Has the patient current medical treatment for angina?	Y / N	
• Has the patient current medical treatment for heart failure?	Y / N	

Height _______________cm Blood pressure (sitting) ______/______mm Hg

Weight _______________kg

Waist circumference ________cm Right index finger circumference ______mm

counted with: O rings O tape measure

S-IGF-1____________μg/l Reference for age___________μg/l

- Is the patient's acromegaly biochemically controlled? Yes / No
  (S-IGF-1 in the reference range for age)

- **Referred to sleep investigation**:
  - O *Written referral sent at today's visit.*
  - O *No – has previously been investigated for sleep apnea – please see page 1.*
  - O *No – very low clinical suspicion for sleep apnea at today's visit.*

Is there a referral response from the sleep clinic? O No O Yes

Date of investigation _______________

Other information: ____
